# Mutational load and mutational patterns in relation to age in head and neck cancer

**DOI:** 10.18632/oncotarget.11312

**Published:** 2016-08-16

**Authors:** Stefano Meucci, Ulrich Keilholz, Ingeborg Tinhofer, Oliva A. Ebner

**Affiliations:** ^1^ Charité Comprehensive Cancer Center, Charité University Hospital, Charitéplatz, Berlin, Germany; ^2^ Department of Radiation Oncology and Radiotherapy, Charité University Hospital Berlin, Translational Radiation Oncology Research Laboratory, Charitéplatz, Berlin, Germany

**Keywords:** head and neck squamous cell carcinoma, aging, somatic mutations, sequencing, genomics, Gerotarget

## Abstract

Head and neck squamous cell carcinoma (HNSCC) is a cancer with well-defined tumor causes such as HPV infection, smoking and drinking.Using The Cancer Genome Atlas (TCGA) HNSCC cohort we systematically studied the mutational load as well as patterns related to patient age in HNSCC. To obtain a homogenous set we excluded all patients with HPV infection as well as wild type TP53. We found that the overall mutational load is higher in patients of old age. Through unsupervised hierarchical clustering, we detected distinct mutational clusters in very young as well as very old patients. In the group of old patients, we identified four enriched pathways (“Axon Guidance”, “ECM-Receptor Interaction”, “Focal Adhesion” and “Notch Signaling”) that are only sporadically mutated in the other age groups. Our findings indicate that the four pathways regulate cell motility, tumor invasion and angiogenesis supposedly leading to less aggressive tumors in older age patients. Importantly, we did not see a strict pattern of genes always mutated in older age but rather an accumulation of mutations in the same pathways. Our study provides indications of age-dependent differences in mutational backgrounds of tumors that might be relevant for treatment approaches of HNSCCs patients.

## INTRODUCTION

Head and neck squamous cell carcinomas (HNSCCs) affect 600,000 patients per year worldwide [1]. HNSCCs are characterized by phenotypic, etiological, biological and clinical heterogeneity and can originate from the paranasal sinuses, nasal cavity, oral cavity, pharynx and larynx. The major known risk factors of HNSCC are consumption of tobacco and alcohol, as well as human papillomaviruses (HPV) infection [2]. Multiple studies have elucidated the specific genetic background of HNSCC, establishing subclasses of tumors alongside HPV infection and/ or TP53 mutations. Due to the heterogeneity of study cohorts, the estimated percentages are relatively high variable. Overall, approximately 20% of HNSCCs contain transcriptionally active human papillomavirus (HPV+), and mainly TP53 wild type, which however is inactivated by the viral E6 and E7 oncogenes [3]. The incidence in HPV positive tumors, in oropharyngeal tumors, is exceeding 50% in current cohorts [4–6], and these tumors have been associated with a favorable clinical outcome [7, 8]. Approximately 80% of HNSCCs are HPV-negative (HPV-), themajority of them contain a mutation in TP53 and are characterized by many numerical geneticchanges (high chromosome instability). In the remaining cases, characterized by a lower number of numerical genetic changes, p53 seems not to be inactivated [3].

Tumor characteristics vary between patients of different ages. Elderly patients are mainly diagnosed with a lower incidence of regional lymph node metastasis at diagnosis, often associated with a less aggressive tumor phenotype [9, 10]. Yet, whether older patients have similar or shortersurvival is up for debate and shows controversial results in different studies [11–13]. Ageing related physiological alterations and the duration of active smoking should be simultaneously taken into account. As shown in lung cancer the onset of smoking is usually in adolescence or young adult age, and smoking cessation is associated with the diagnosis of the malignancy [14,15]. In addition, several studies report HPV-negative HNSCCs mostly occurring in smokers [16–18]. Therefore the age at diagnosis of HNSCC patients is very closely correlated to the duration of smoking.

While some differences in tumor behavior in older patients have been recorded, no study so far systematically explored the relationship between genetic tumor background and age in HNSCC. A recent study performed on the extensive data set available on The Cancer Genome Atlas (TCGA) portal showed the age-related accumulation of somatic mutations in diverse human tissues [19]. However, it is still an open question whether differences in mutations between the ages are random coincidence or follow distinct patterns. Therefore, this study aims to explore the specific age-mutation relationship in tumors of HNSCC patients to determine if age-related genetic parameters have to be considered in the disease prognosis and treatment decision.

To investigate a possible connection between patterns and frequency of genetic mutations and patient age, we used the recently published TCGA study on HNSCCs of 279 patients.

HPV infection shows an age bias as a relevant impact on the mutational background of the tumor. In previous studies the mutation rate of HPV-positive tumors was lower than that found in HPV-negative HNSCC, consistent with recent epidemiologic studies that establish biological differences between HPV-positive and HPV-negative disease [20]. The major biologic difference between HPV-positive and - negative tumors, however, concerns p53, which in its role as a guardian of the genome influences multiple genes. In general, patients with HPV-positive tumors have non-mutated TP53, however, HPV itself inhibits p53 function. Conversely, HPV-negative tumors frequently harbour TP53 mutations.

Thus, in order to select a homogenous patient cohort for investigating possible age-related differences, we only considered the 203 patients from the TCGA-cohort which were HPV negative and carried a TP53 mutation. This homogenous subset of patients allowed for a systematic study of the age influence on mutation load and spectrum without introducing a heterogeneity caused by HPV or p53. Of course, it would have been equally interesting to study the other subclasses of HNSCCs. However, since only few patients belonged into these subsets, a statistical sound investigation would not have been possible within the current TCGA cohort.

## RESULTS

### Patients selection and cohort features

**Figure 1:**
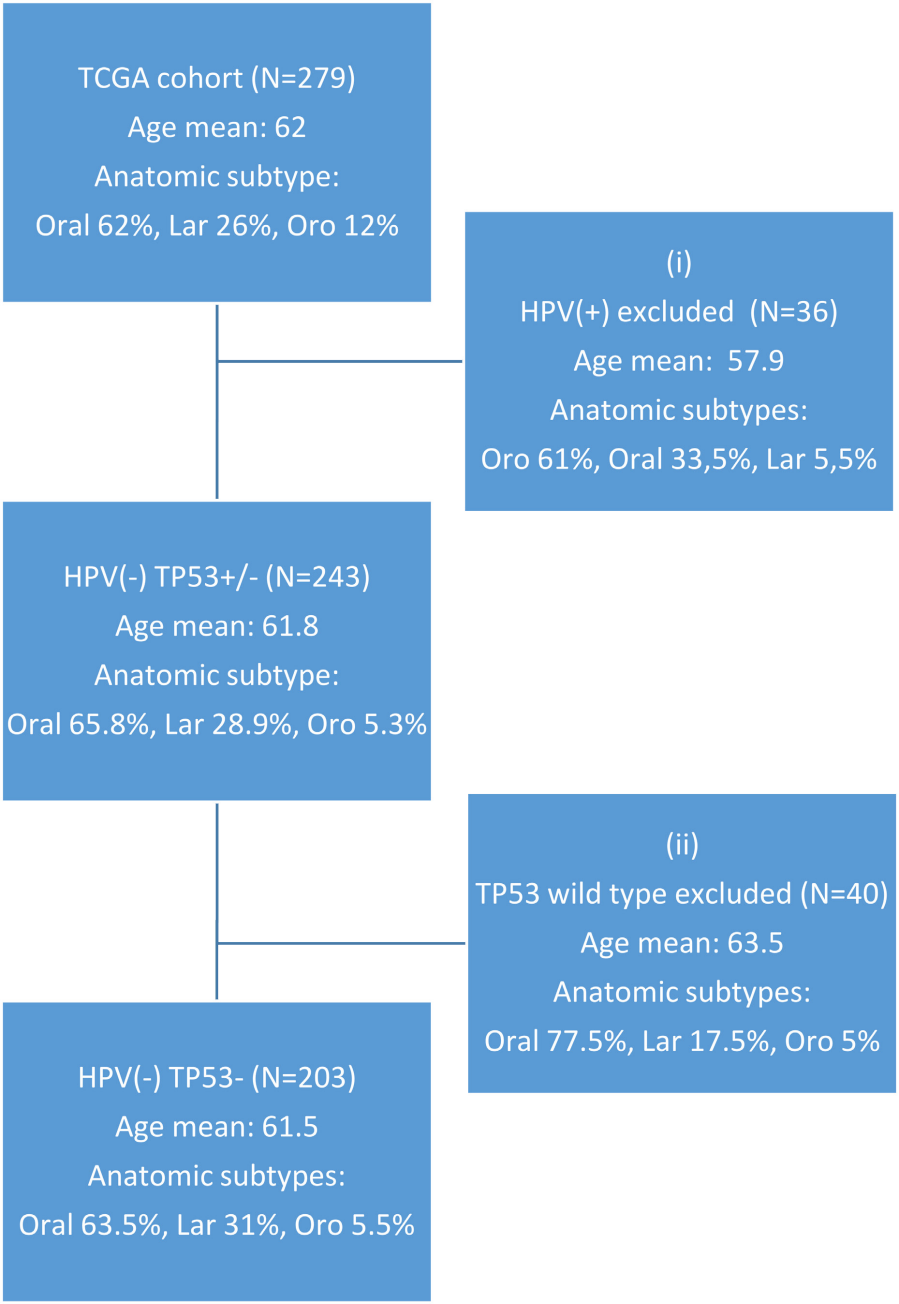
CONSORT diagram of original and selected patient cohort

To create a homogenous set of tumors we used a subset of the TCGA cohort (Figure 1). The original patient cohort was selected by two criteria, the first (i) excluded 36 HPV(+) patients (HPV classification according to the TCGA publication). As expected, the HPV-positive phenotype was strongly associated with the oropharyngeal site and the patient age mean was lower than for the HPV-negative patients. The second selection criteria (ii) excluded 40 TP53 wild type patients, which left the final selectedcohort of 203 HPV-negative/TP53-mutated patients. The original TCGA data set showed a higherrate (86%) of TP53 mutations among HPV-negative samples than have been previously reported, while only 1 out of 36 HPV-positive cases had a non-synonymous TP53 mutation. Our selection rendered 203 patients with a total amount of 29.860 single nucleotide polymorphisms (SNPs)distributed on 11.489 genes.

Statistical hypothesis tests showed that the patient selection does not lead to a biased distribution of patients features such as age, smoking and alcohol consumption. Whereas, thehypergeometric test performed on the tumor localization distribution showed an enrichment inLarynx tumor (*p* < 0.05) and a depletion in Oropharynx tumor (*p* < 0.05) in the selected cohort (Table S1).

A comparison of the original TCGA cohort and our subset can be found in Table 1.

Furthermore, the results of the investigation of TNM and overall staging at diagnosis in relation to the age showed a smaller size of the primary tumor, a decrease of lymph node metastasis and a higher percentage of “localized cancers” and “first stageof locally advanced cancers” in old patients (Table S1). The youngest patients group showed the same statistics as the oldest, however the results are not comparable due to the significant difference between the two age group ranges.

**Table 1:** Comparison of TCGA cohort and selected patients

Characteristics	TCGA (279 patients)	Selected cohort (203 Patients)
*Age (years)*		
Median	61	62
Range	19-90	19-87
*Smoking history*		
Yes	220	159
No	52	37
Unknown	7	7
*Primary tumor location*		
Oral Cavity	172	129
Larynx	72	64
Oropharynx	33	9
Hypopharynx	2	1
*HPV/ p53 status*		
HPV + / p53 +	35	0
HPV + / p53 -	1	0
HPV - / p53 +	40	0
HPV - / p53 -	203	203

### Increase of mutation frequency with age

When looking at the mutations in each patient, a wide range in numbers along with a single extreme value in one patient (age 69) was observed (Figure S1).

**Figure 2:**
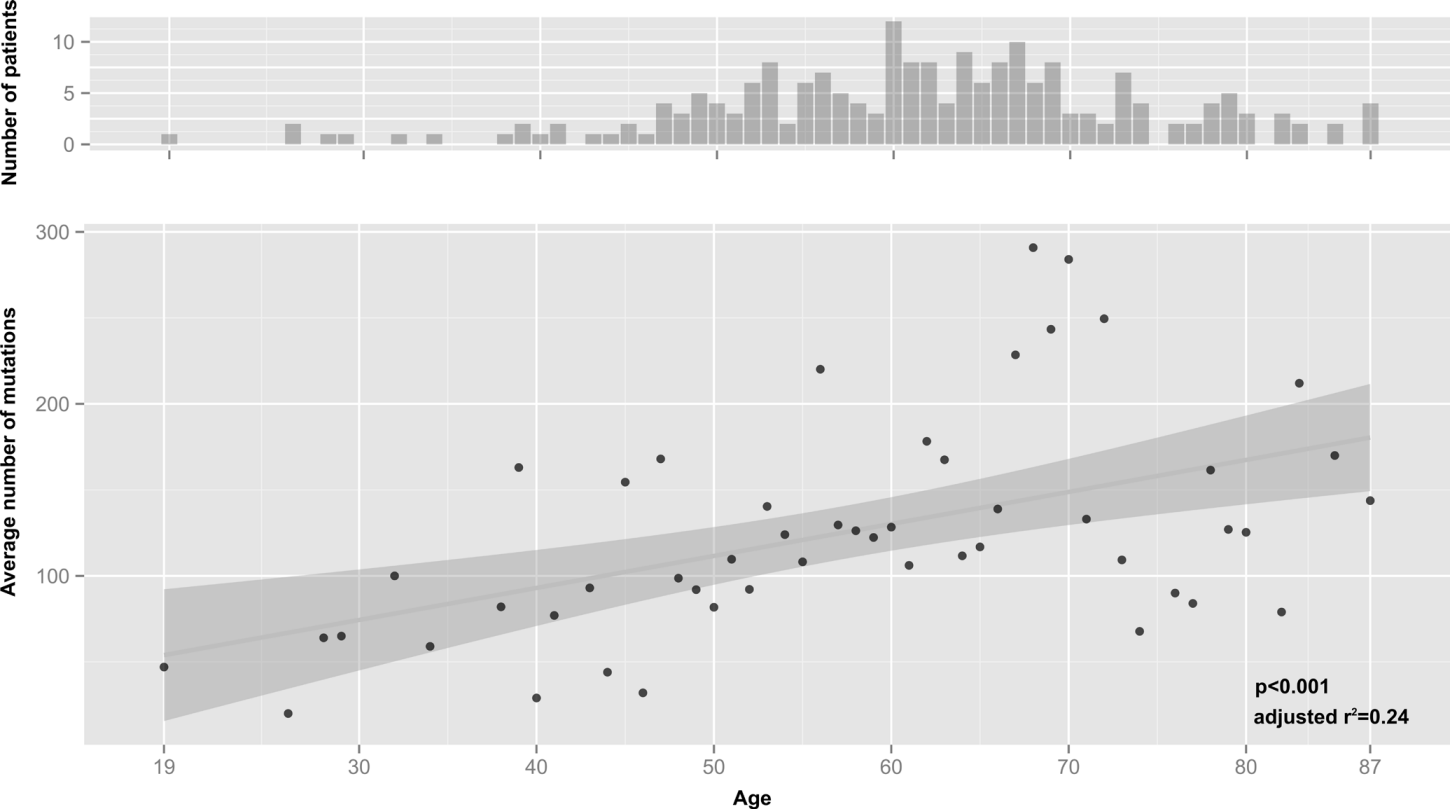
Correlation of average mutations and age considering one mutation per gene Upper graph number of patients in the respective age group. Lower graph, average number of mutations for all patients in the respective age groups. Linear regression analysis was done using F-statistics and shows a significant increase in mutations in older patients (*p* = 0.000161, adjusted r^2^ = 0.24). Grey area around regression line indicates 95% confidence interval. Regression without the patient having an extreme number of mutations (TCGA-D6-6516, see Figure S1) also yields a significant connection between average mutations and age *p* = 0.000291, r^2^ = 0.22 (data not shown).

A significant rise (*p* < 0.01) in the average number of mutations in different genes was observed with increasing age (Figure 2), which was also true if the patient with the extreme number of mutations was removed. Interestingly, we repeated the mutation load analysis excluding silent mutations and multiple mutations in one gene, to have an additional overview of disruptive mutations with increasing age. Although the number of mutations in each patient decrease drastically, the same significant p-value was detected (Figure S2, list of mutation frequencies in Table S2). Thus, the number of mutated genes found in a tumor was higher in older patients than in younger patients, designating a quantitative difference in mutational load between patient ages irrespective of whether this signified a pure stochastic increase or the accumulation of disease relevant mutations. Interestingly, there were only five genes with recurrent mutations, three of which are well known players (TP53, CDKN2A, PIK3CA) and two are pseudogenes (RPSAP58, WASH3P).

In order to investigate the influence of patients features such the tumor localization and the smoking consumption on the increasing of the average number of mutations with age, we repeated the regression analysis for each subset. Although it's necessary to consider the different number of patients in each subset and the difficulty on the acquisition of smoking habits (possible false-positive/-negative), both smokers and non-smokers subsets showed a significant correlation (linear regression as well as Spearman's rank correlation analysis), therefore the smoke history surprisingly seems not to influence the age-related mutational load of our sub-cohort. Whereas, the mutational load of our sub-cohort as well as the original cohort are mostly influenced by the location of the cancers, only oral cavity tumors showed a significant correlation with age (Table S1).

### Relationship of mutational patterns and age

As a next step, we wanted to investigate whether this increase was a mere accumulation of random mutations or whether we could find age-specific patterns of mutated genes. Unsupervised hierarchical clustering based on gene mutation frequencies was performed for patients pooled into age groups of decades aside two groups of the very young (pooled age 19-40) and very old (pooled ages 81 to 87). A minimum difference of 0.15 in mutation frequency between at least two age groups was considered for cluster analysis (Figure S3). This cut-off allowed for a comprehensive set of mutational patterns within the specific age groups including less frequently mutated genes.

**Figure 3:**
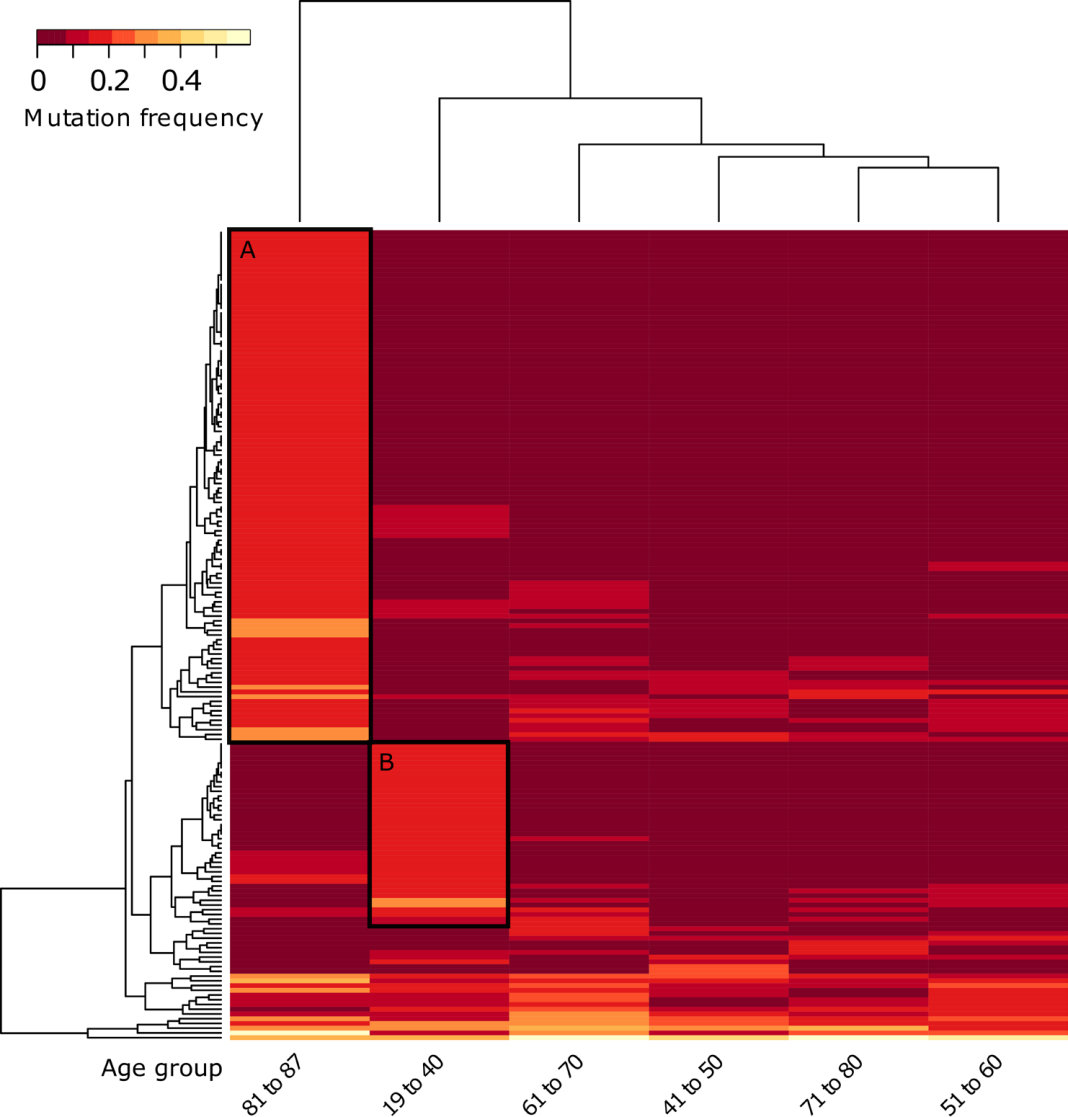
Unsupervised hierarchical clustering of gene mutation frequencies of specific age groups with pooled young and old ages Patients were grouped into age groups of very young (ages 19‐40) and very old (ages 81‐87) with 10‐year bins in between, then clustered according to the mutations frequencies of the quantified genes. Only genes with a minimum frequency difference of 0.15 between at least two of the age groups are displayed. Black boxes indicate extracted genes for **A.**, age group 81 to 87 and **B.**, age group 19 to 40 (for genes see Table S3).

The results showed two relevant clusters of frequently mutated genes, in particular, 39 genes in very young patients and 108 genes in very old patients (Table S3 with cut clusters A and B from Figure 3, unmarked heatmap Figure S3). Both the young as well as the old group, each consisting of 11 patients, displayed prominent differences to middle age groups. The genes of the two specific clusters were tested for KEGG pathway enrichments using the online David Gene Ontology tool [21]. The old age gene cluster showed six statistically significant enriched pathways, two of which, the “Axon Guidance” (*p* < 0.003) and “ECM-Receptor Interaction” (*p* < 0.04) pathways, stood out due to their role in angiogenesis processes and cell / tissue architecture maintenance respectively (Table S4).

Six “Axon Guidance” genes (SEMA5A, DCC, PLXNB2, UNC5D, ITGB1, EPHA2) and four of the “ECM-Receptor Interaction” pathway (LAMA2, LAMA4, TNC, ITGB1) were significantly enriched. In contrast, no enriched pathways were found for the young age gene cluster. To see if the very old and very young patients were homogenous groups or comprised of smaller subgroups we looked at both in more detail. Further division of the young age group into two fractions comprising ages 19 to 35 (7 patients) and 36 to 40 (4 patients) did yield an enrichment of the TGFB pathway for the latter age group (Figure S3, Table S4). This enrichment, however, was mainly based on the mutations of the enriched genes in one single patient (age 39). In addition, TGFβRII and TGFb1, two main players of the TGFB pathway and responsible for more aggressive tumors in HNSCCs were not mutated in the young ages at all [22]. We therefore did not interpret this finding as an age-specific enrichment.

### Old age specific clusters and pathway enrichment

The old age group, on the other hand, showed distinct clusters and enrichment when split into smaller fractions of ages 81 to 85 (7 patients) and 86 to 87 (4 patients, all age 87).

**Figure 4:**
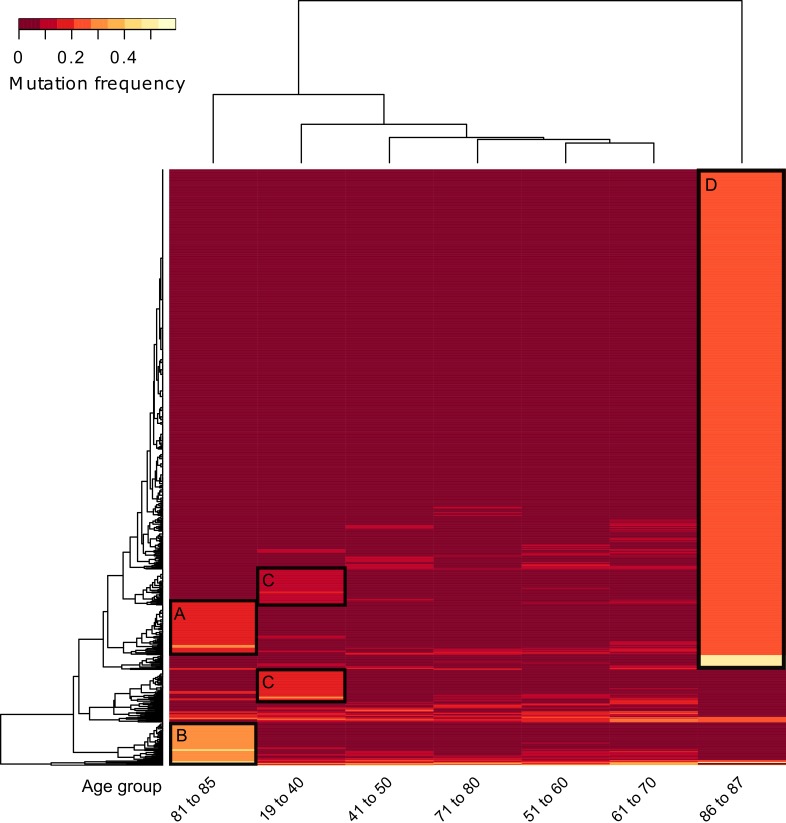
Unsupervised hierarchical clustering of gene mutation frequencies of specific age groups with separate old ages Patients were grouped into age groups of very young (ages 19‐40), decades in-between and two separate old ages groups (ages 81‐85 and 87), then clustered according to the mutations frequencies of the quantified genes. Only genes with a minimum frequency difference of 0.15 between two of the age groups are displayed. Black boxes indicate extracted genes for **A.**, age group 81 to 85 upper cluster (shared with ages 86 to 87), **B.**, age group 81 to 85 lower cluster (separate genes from ages 86 to 87), **C.**, two clusters of ages 19 to 40 and **D.**, ages 86 to 87 (for genes see Table S3).

Figure 4 shows a major cluster of 542 genes in the “87” age class (Cluster D) and two gene clusters in the “81‐85” age group (Cluster A and B, unmarked heatmap Figure S5). The latter consisted of 47 specific mutations and 59 genes overlapping with the “87” age group, indicating a common genetic background of tumors in elderly patients (Table S3).

Note that we included six genes from the old ages clusters (CDKN2A, CSMD3, FAT1, NOTCH1, PIK3CA, TTN) that did make the 0.15 cutoff, but were in fact frequently mutated in most other ages as well. However, since the enrichment analysis showed significant p-values for both the set with as well as without the six genes we kept them in our gene set.

The “87” age group showed six significantly enriched pathways among which are again “ECM-Receptor Interaction” (*p* < 0.006, 11 genes) and “Axon Guidance” (*p* < 0.006, 13 genes), and in addition “Notch Signalling” (*p* < 0.007, 7 genes) as well as “Focal Adhesion” (*p* < 0.05, 15 genes) (Table S4). The 47 genes specifically mutated in the “81‐85” group did not yield any enrichment. However, when combining all highly mutated genes in this group, “Axon Guidance” was enriched as well yet with a slightly elevated p-value (*p* = 0.056, 4 genes) (Table S4). The overlap of the two old age groups is only three genes (PLXNB2, SEMA5A, UNC5D), yet genes of the same families (such as Ephrins, Slits and Rho-associated protein kinases) were mutated in both age groups. Overall, the old age groups only revealed a certain degree of homogeneity, with the very old patients (age 87) showing distinct pathway enrichment. However, the high number of overlapping genes as well as the commonly enriched pathways fostered the idea of specific mutational trends happening at the old age rather than one specific mutational pattern.

### Mapping of “Axon Guidance” pathway genes to all age groups

**Figure 5:**
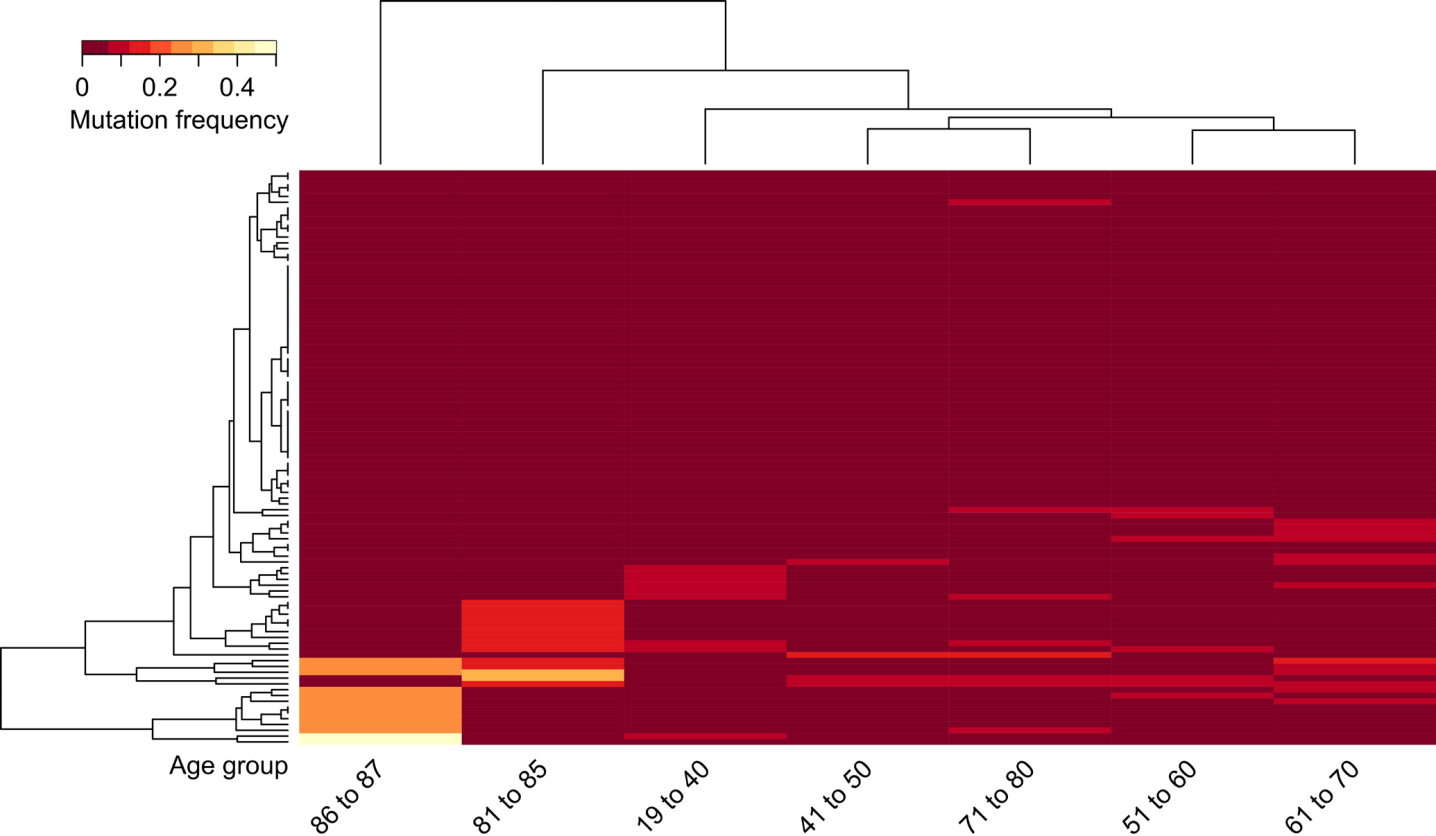
Unsupervised hierarchical clustering of mutation frequencies of genes involved in the “Axon Guidance” pathway (according to the KEGG database) Patients were grouped into age groups of very young (ages 19‐40), decades in-between and two separate old ages groups (ages 81‐85 and 86 to 87), then clustered according to the mutation frequencies of all quantified genes of the “Axon Guidance” pathway. Genes that did not make the original cut off of 0.15 difference in mutation frequency but are found mutated in this pathway in age group 81‐85 are LRRC4C, DPYSL2, EPHA8, LIMK1, NCK1, NGEF, RAC1, ROBO2, ROCK1 and SLIT2.

We mapped all genes of the “Axon Guidance” pathway (according to the KEGG database) to our list of mutation frequencies to see if the pathway is affected in other age groups as well without being particularly enriched (Figure 5, Table S5). From the total 127 genes of the “Axon Guidance” pathway 99 mapped to our data. As the heatmap showed, mutations in this pathway were present in other ages as well, yet at a lower frequency than in the two old age groups. Again, the trend of increased pathway alterations towards old ages was visible. In addition to genes mutated at a high frequency we found a group of 10 genes mutated at lower frequencies in the “81‐85” group (Figure 5).

### Pathway enrichment upon fusion of clusters

As the mapping of all genes to the “Axon Guidance” pathway had shown, we were missing mutated genes of a pathway that did not make the 0.15 frequency cutoff. However, since our results suggested that mutations in old patients did not necessarily follow a strict common pattern and different genes were affected, we did not expect all mutations in a pathway to occur at high frequencies. At the same time we had seen a common trend for both old age groups with impact of mutations on similar gene families. Thus we decided to merge the highly mutated genes of both separate age groups for a more comprehensive picture of affected pathways.

Pathway enrichment analysis was performed on a consistent group of 589 mutated genes, many of which were hidden by the 0.15 clustering threshold when calculating the mutation frequency of the two old ages groups together, rather than separately.

The result showed 10 significantly enriched pathways (Table S4), two of which had been found enriched before, namely “Axon Guidance” (*p* < 0.004, 14 genes) and “ECM-Receptor Interaction” (*p* < 0.001, 13 genes). In addition, “Notch Signaling” (*p* < 0.002, 8 genes) and “Focal Adhesion” (*p* < 0.009, 18 genes) were selected for detailed analysis due to their important role on cell growth, cell / tissue architecture and cell motility. When mapping all pathway genes to our data, we saw in all three the same pattern as in the “Axon Guidance” pathway of sporadic mutations that occur in all ages, yet the frequency of mutated genes was much higher in the old age groups of 81 to 87 (Figure S4-S6, Table S6–S8).

Overall, we identified 24 genes mutated in the “Axon Guidance” pathway among the 81‐87 group of patients. 14 genes resulted from the pathway enrichment analysis and 10 genes were additionally revealed by the pathway heatmap clusters (Table S9). Even though 79% (19 out of 24) carried a single mutation showing the tumor heterogeneity, in the end 82% of the old patient group “81‐87” (9 out of 11) reported a disruption in the Axon Guidance pathway. Furthermore, 82% (9 out of 11) of the patients showed mutations in “ECM-Receptor Interaction” pathway and all patients reported mutations in “Focal Adhesion” pathway. In particular we identified 21 and 34 significantly mutated genes respectively, which overlapped in 20 genes (Table S9). Interestingly we found several mutated genes of the same protein families, six laminins (LAMA1, LAMA2, LAMA3, LAMA4, LAMC1, LAMB1), three integrins (ITGA11, ITGA2, ITGB1) and eight collagen mutate genes (COL1A1, COL11A1, COL29A1, COL6A3, COL27A1, COL11A1, COL4A5, COL4A4), crucial in ECM-signaling processes and focal adhesions, as well as six ephrin genes (EPHA2, EPHA3, EPHA5, EPHA6, EPHA8, EPHB6), which have a central role in “Axon Guidance” signalling processes. Lastly, 64% of the patients (7 out of 11) displayed mutations in “Notch-Signaling” pathway. We found 10 mutated genes, three of which were NOTCH genes (NOTCH1, NOTCH3, NOTCH4).

Altogether, we only saw a few genes recurrently mutated and thus no age specific mutational pattern. However, even though not always the same genes were affected, the mutations accumulated in the same pathways, indicating a common trend in elderly patient to have similar functions of the cell changed by mutations.

## DISCUSSION

In the present study, we evaluated the somatic DNA single nucleotide polymorphisms (SNPs) landscape of a selected HNSCC patient cohort in relation to ageing processes. The average number of mutations for each patient showed a significant rise (*p* < 0.01) with increasing age. Multiple factors participate to the accumulation of genetic eventsin the elderly such as prolonged tobacco exposure and ageing-related genomic instability. To provide better insight into the underlying mechanisms, we therefore investigated whether amere accumulation of random mutations or distinctive mutational patterns are related to patient age.

Unsupervised clustering of the selected cohort showed distinct clusters of genes expressed with a higher mutation frequency for the very young and very old ages. While the young age group did not reveal pathway enrichment, the old age group showed enrichment of KEGG pathways, including “ECM-Receptor Interaction” and “Axon Guidance”. Further division of the old age group into ages 81 to 85 and age 87 showed several genes of the same pathways shared by the two groups. Which when clustered together showed ten enriched pathways. Besides “Axon Guidance” and “ECM‐Receptor Interaction”, “Notch‐Signaling” and “Focal Adhesion” were of special interest to us. When mapping all genes of the respective pathways to our data, we saw mutations in all patients, however no evident clusters were present other than for ages 81 to 87. These results indicate that while the specific mutational patterns might only exist in very subtle ways due to the heterogeneity of the tumors, we could see an increase in mutations in distinct pathways over the ages with a peak in the very old fraction. Next, we looked at the involved genes in more detail, starting with the “Axon Guidance” pathway.

Although “Axon Guidance” represents a key stage in the formation of neuronal networks, recent studies also linked this pathway to regulation of angiogenesis processes (endothelial cell migration, proliferation and vessel formation). Involved proteins are Netrins, Slit proteins, Semaphorins, Ephrins and their cognate receptors (e. g. UNC5, ROBO1-4), all of which are frequently mutated in the elderly groups [24,25]. The expression of Eph receptors, five of which were mutated in our data, is frequently elevated in different types of malignant tumors possibly resulting in increased cellular motility, tumor cell invasion and metastasis [26, 27]. One of these, EPHA2, is overexpressed in squamous cell carcinoma of oral tongue [27] and was also protruding in our mutational analysis. Moreover, studies have proven that blocking EphA receptor signaling decreases tumor vascular density, volume and cell proliferation *in vivo* [28–31]. Since we found many of these genes to be mutated in the old age group, we postulated a correlation between “Axon Guidance” aberrations and the HNSCC features of elderly patients which may result in decreased cellular motility and tumor cell invasion.

For the “ECM‐Receptor Interaction” pathway, we identified 21 frequently mutated genes, including six laminins, three integrins and eight collagens. These molecules are structurally and functionally involved in interactions at the extracellular matrix (ECM) which lead to a direct or indirect control of cellular activities such as cell migration, differentiation, proliferation, and apoptosis [32, 33]. A re‐expression of the laminin α2 and α4 chains, which were significantly mutated in our old patients group, could be shown in adult hyperproliferative, dysplastic and carcinomatous lesions [31, 32]. Several studies showed Laminin 332 (LAMA3, LAMB3 and LAMC2) to be highly expressed in HNSCC and foster tumor invasiveness, an effect that is reversed when the laminins are repressed by microRNA‐29s [36–40]. Several other integrins composed by the Integrin β1 chain, highly mutated in patients of old age, have been identified as crucial for tumor cell invasion and angiogenesis [38, 39]. Altogether, the many mutations in this pathway again suggested a decreased cell migration and tumor invasion in old patients.

The “Focal Adhesion” pathway was significantly enriched in the 81‐87 age group as well. We identified 34 genes, 20 of which overlap with the “ECM-Receptor Interaction” pathway, in particular laminins and integrins. The latter regulate kinases, such as the focal adhesion kinase (FAK), which is crucial for the attachment and signal transduction between cells and the ECM [43]. Several studies demonstrated that FAK disruption caused decreased cell attachment and motility while FAK overexpression increased cell invasion in HNSCC [41, 42]. Therefore mutations on upstream proteins like laminins and integrins again suggested a decreased cellular motility in HNSCC.

NOTCH signalling is a highly conserved pathway that plays distinct roles during tissue homeostasis, proliferation and apoptosis [46]. Even though NOTCH1 is one of the most frequently mutated genes in HNSCC, there are contradictory studies about its influence on tumor development [20]. Loss-of-function mutations in the NOTCH1 gene have been detected in a significant proportion of patients [47]. On the other side it is known that NOTCH activation can enhance proliferation, inhibit apoptosis and promote angiogenesis [45, 46]. Thus, while we reported high mutation frequencies for 10 genes of the notch pathway (inter alia NOTCH1, NOTCH3, NOTCH4), we couldn't make assumptions about the exact impact of this finding.

Concluding, we found a proportional increase of the mutation frequency rate in relation to the age of the HNSCC patients. This increase, however, did not follow distinct mutational patterns but rather an accumulation of mutations in specific pathways.

The results of this pathway analysis suggested a reduced tumor invasiveness and metastasis in older patients, which was underlined by the tumor staging at diagnosis. This distinct mutational background might be relevant for treatment approaches decisions and should therefore be taken under closer consideration in future studies.

## MATERIALS AND METHODS

### Acquisition and processing of data

The clinical data of the TCGA patient set was derived via download from the TCGA data matrix (https://tcga-data. nci. nih. gov/tcga/dataAccessMatrix. htm). Somatic mutations of HNSCCs from the TCGA study was derived by download from the cBio Portal [18, 19]. The somatic mutations of the 279 HNSCC patients detected by exome sequencing within the TCGA project [16] were merged with the clinical data from TCGA to combine genomic mutations with the age information of all patients. Entries without official gene names were removed. Since we were interested in patients with a HPV negative genomic background, we excluded all patients with a positive HPV status according to the standards of the TCGA publication. As HPV determination has certain limitations [16], it remained uncertain, whether all tumors classified as HPV negative were truly negative, or whether diagnostic sensitivity may have misclassified some cases. Therefore, we selected for patients with at least one mutation in TP53 for our study, as TP53 mutations are generally not found in HPV positive tumors. For a first evaluation of the mutational rate related to age we considered all mutations, including silent mutations as well as multiple mutations in one gene. For all subsequent analysis, silent mutations were removed and multiple mutations in the same gene were only considered once per patient to prevent statistical overestimations.

### Statistical analysis

**Mutation frequency and age of patients**

The primary statistical hypothesis was, that there would be an increase in the number of mutations with increasing age. The relations between total average mutation frequencies and patient age groups (in years) were calculated by linear regression using F-statistics. A two-sided p-value of below 0.05 (Pr(>|t|)) was considered significant. The frequency of mutation of a specific gene was calculated using the sum of mutations in a specific age group divided by the number of patients in the respective age group. Additional Spearman's rank correlation analysis was performed to identify the genes whose mutation frequency correlates with the age (Table S10).

All data analysis was done using R [52] unless stated otherwise.

**Recurrent genes**

To check for recurrent genes we only considered genes mutated in at least five patients with the same starting and end position of the mutation.

**Clustering and pathway enrichment analysis**

Unsupervised hierarchical clustering based on gene mutation frequencies was performed fordifferent age groups. Genes were clustered according to Euclidian distance measure using themethod “complete”. Genes from age group specific clusters were extracted and tested using the online David Gene Ontology tool [21]. The full list of identified genes was used as background for enrichment calculation. In reverse, all genes of an enriched KEGG pathway were mapped to our list of mutation frequencies to investigate the number and distribution of mutated genes in the respective pathway within all ages of our dataset.

## SUPPLEMENTARY FIGURES AND TABLES






















