# Supplementary material for: Mutational load and mutational patterns in relation to age in head and neck cancer
Source: Oncotarget. 2016 Aug 16;7(43):69188–99. doi: 10.18632/oncotarget.11312 (PMC5342469; doi:10.18632/oncotarget.11312)
Supplement: Supplementary file 1 [file oncotarget-07-69188-s001.pdf]

Mutational load and mutational pattern in relation to age in head and neck cancer

Supplementary Material

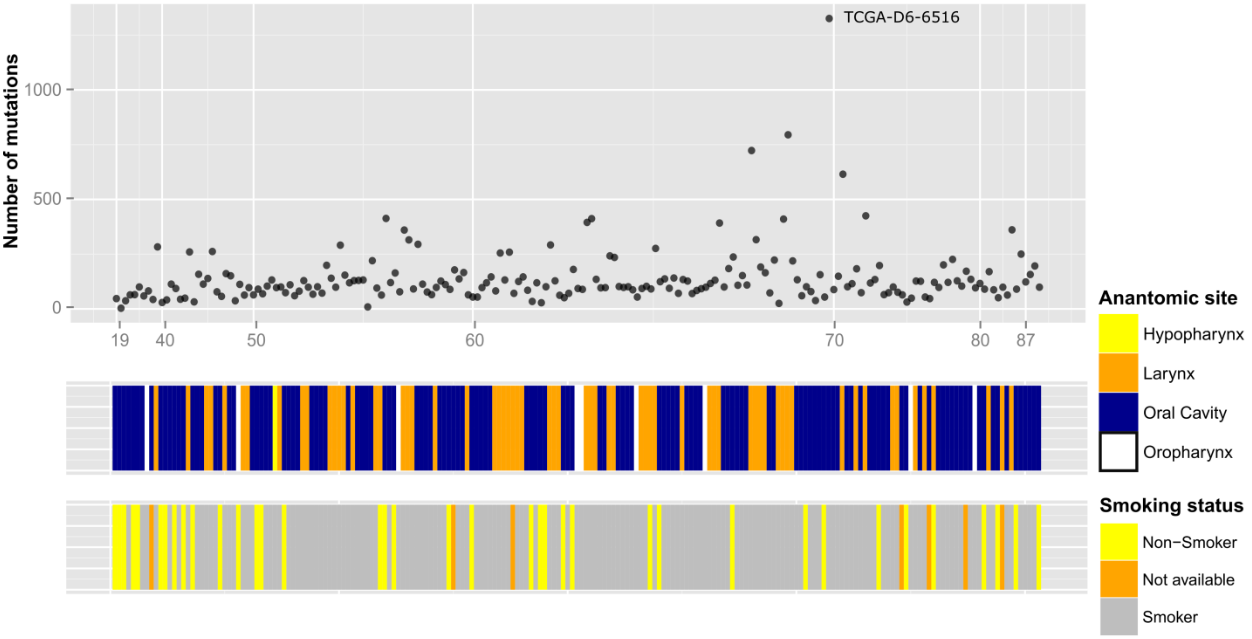

Figure S1: Characteristics of the selected patient cohort

Sum of mutated genes in each of the 203 selected patients ranked according to age and subsequently anatomical site (Sequence Oral cavity, Oropharynx, Hypopharynx and Larynx). Color bars underneath indicate tumor site and smoking habits of the respective patient. Marked outlier patients (TCGA-D6-67516) with extreme number of mutations.

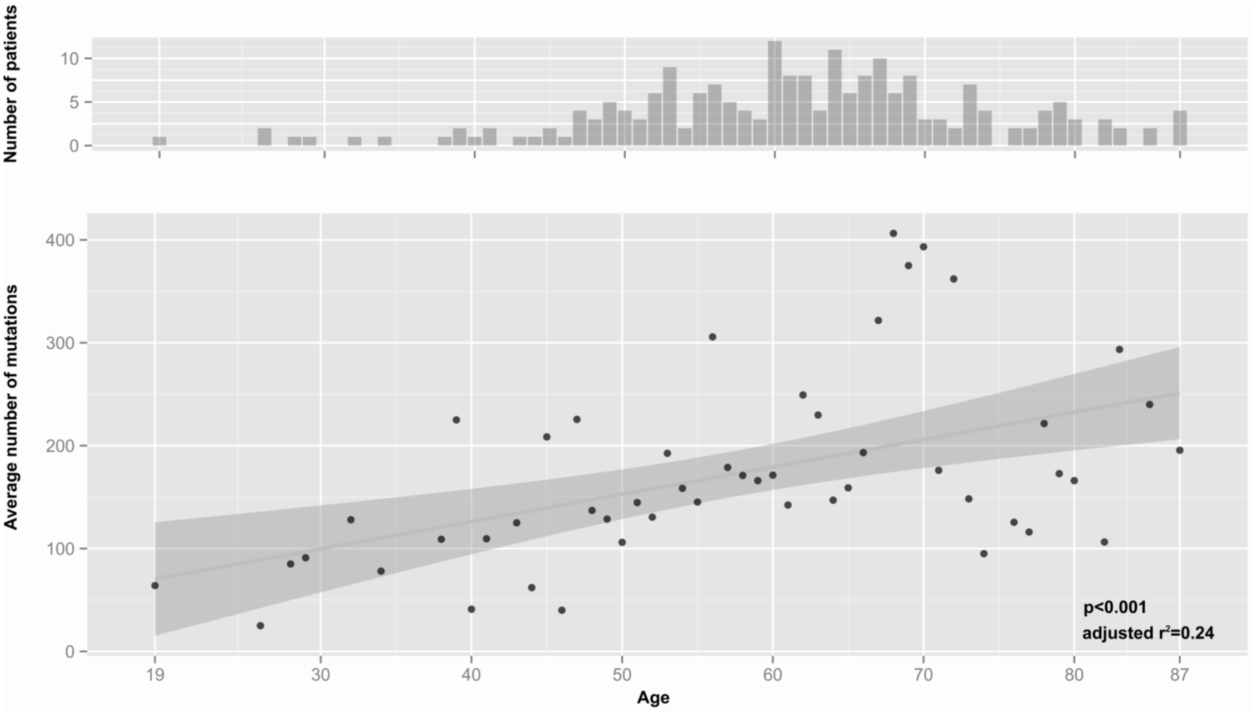

Figure S2: Correlation of average mutations and age considering silent and multiple mutations in one gene

Upper graph number of patients in the respective age group. Lower graph average number of mutations for all patients in the respective age groups. Linear regression analysis was done using F-statistics and shows a significant increase in mutations in older patients (p=0.000179, adjusted r^2=0.24). Grey area around regression line indicates 95% confidence interval. Regression without the one patient having an extreme number of mutations (see Figure S1) also yields a significant connection between average mutations and age p=0.000289, r^2=0.22 (data not shown).

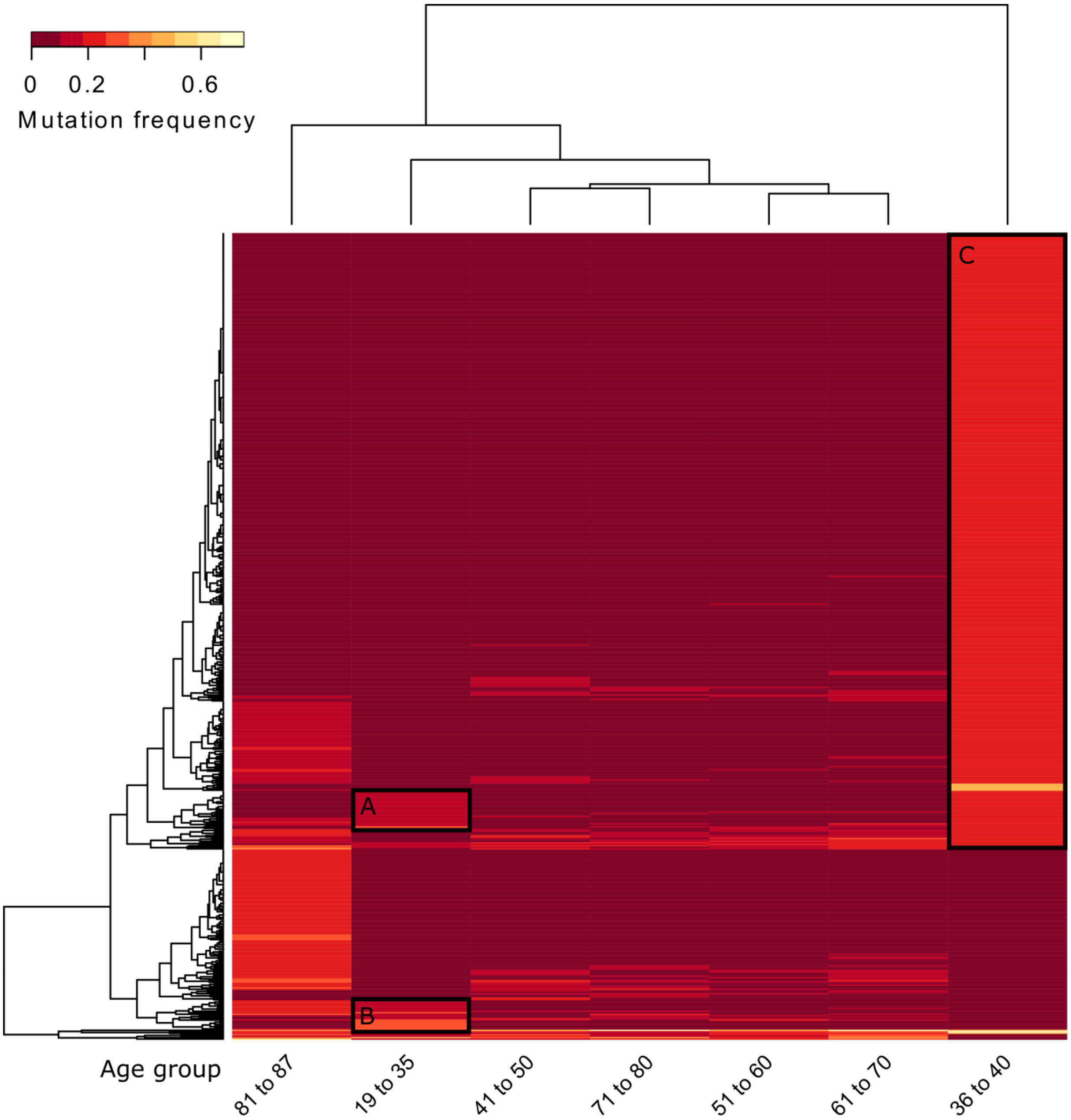

Figure S3: Unsupervised hierarchical clustering of gene mutation frequencies of specific age groups with separate groups for very young ages

Patients were grouped into age groups of decades and very old ages very old ages (ages 81-87). Very young patients were grouped into two separate subsets of ages 19 to 35 and ages 36 to 40. Subsequently, all groups were clustered according to the mutations frequencies of the quantified genes. Only genes with a minimum frequency difference of 0.15 between two of the age groups are displayed.

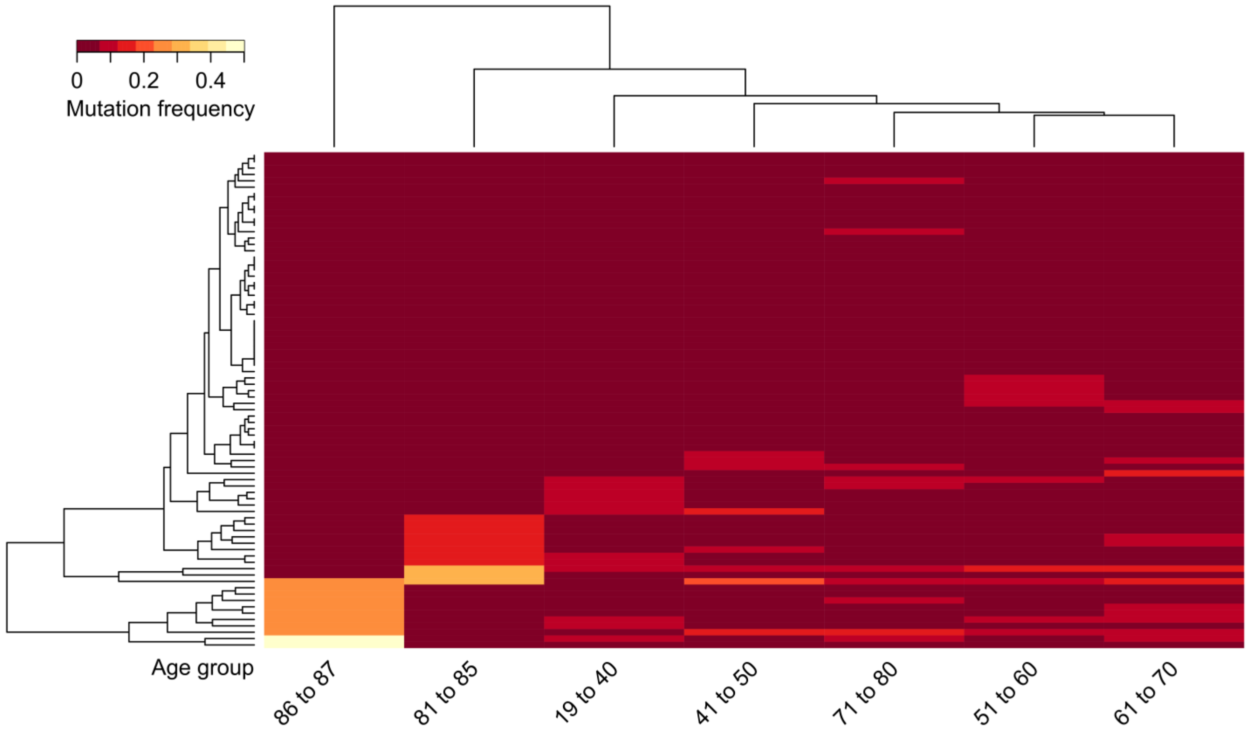

Figure S4: Unsupervised hierarchical clustering of mutation frequencies of genes involved in the "ECM-Receptor Interaction" pathway (according to the KEGG database)

Patients were grouped into age groups of very young (ages 19-40), decades in-between and two separate old ages groups (ages 81-85 and 87), then clustered according to the mutation frequencies of all quantified genes of the "ECM-Receptor Interaction" pathway.

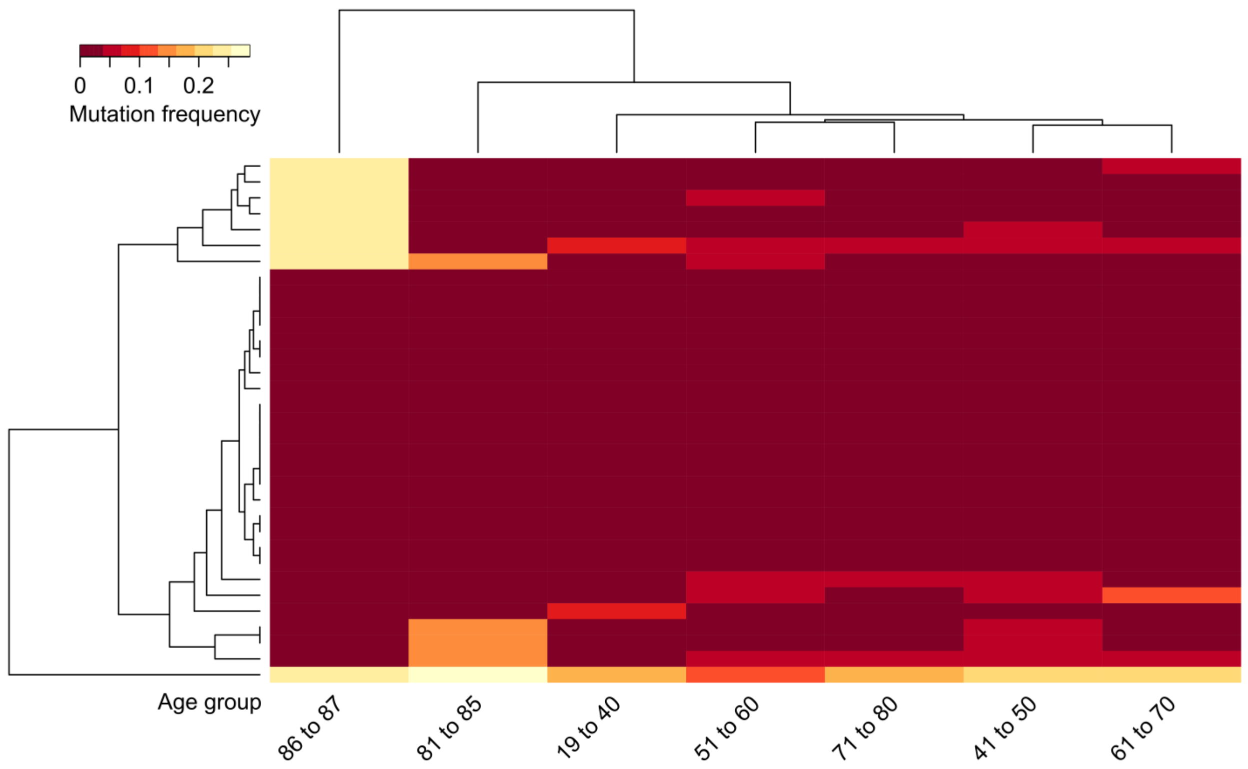

Figure S5: Unsupervised hierarchical clustering of mutation frequencies of genes involved in the "Notch Signaling" pathway (according to the KEGG database)

Patients were grouped into age groups of very young (ages 19-40), decades in-between and two separate old ages groups (ages 81-85 and 87), then clustered according to the mutation frequencies of all quantified genes of the "Notch Signaling" pathway.

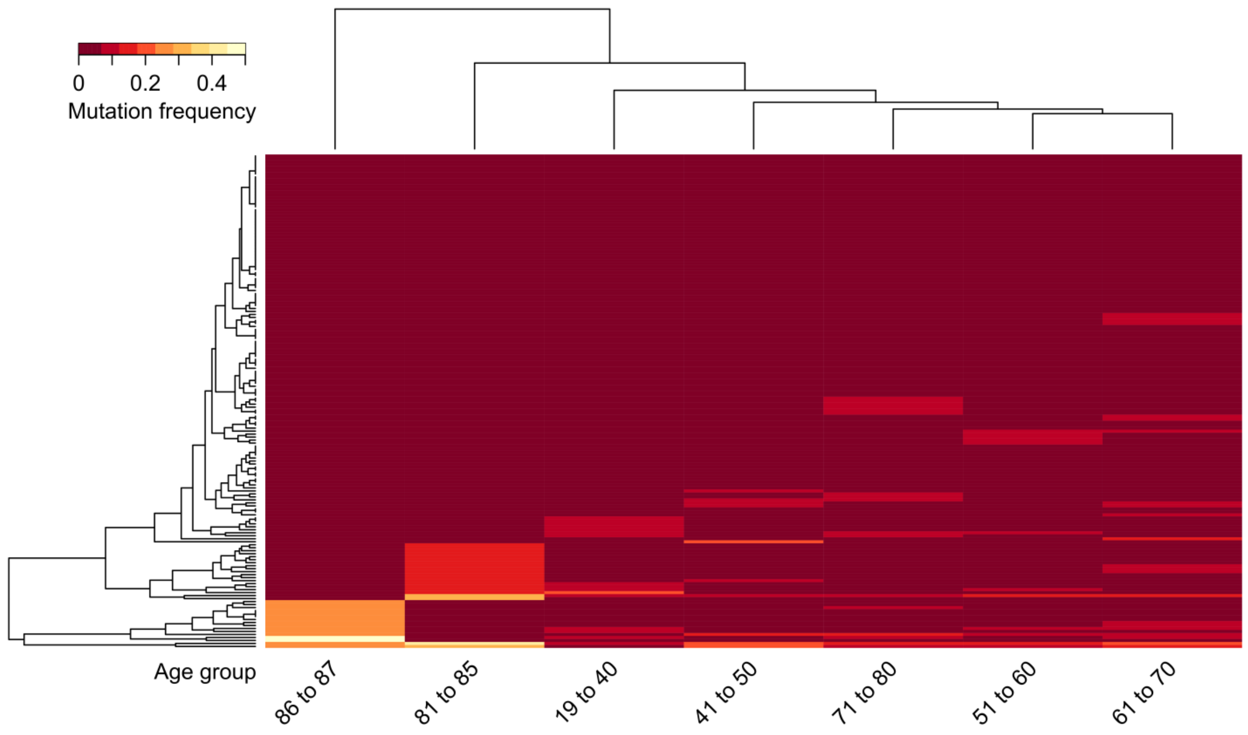

Figure S6: Unsupervised hierarchical clustering of mutation frequencies of genes involved in the “Focal Adhesion” pathway (according to the KEGG database)

Patients were grouped into age groups of very young (ages 19-40), decades in-between and two separate old ages groups (ages 81-85 and 87), then clustered according to the mutation frequencies of all quantified genes of the “Focal Adhesion” pathway.

Supplemental tables

Table S1: Statistical analysis of potential study confounders; mutational load analysis of patient subgroups; TNM and overall staging evaluation at diagnosis in relation to the age

Statistical test were performed to see if our selected cohort was biased towards a certain age (t-test), alcohol or smoking habits (chi-test) or specific tumor locations (hypergeometic test). In addition, the correlation between age and mutational load was tested for given subgroups of the selected cohort (Regression analysis as well as Spearman's rank correlation). The third section shows the statistics on the TNM and overall staging at diagnosis of each age group.

Table S2: List of mutation frequencies in the different age groups (by year) without silent and multiple mutations in one gene

Table S3: Genes extracted from clusters

All the genes extracted from the cluster analysis as well as the IDs used as background for the David Gene Ontology enrichment analysis.

Table S4: KEGG pathway enrichment analysis for old age groups

Results of the DAVID Gene Ontology enrichment analysis for different gene sets as defined in the table.

Table S5: Mutation frequencies of Axon Guidance genes mapped to our data

Table S6: Mutation frequencies of ECM-Receptor Interaction genes mapped to our data

Table S7: Mutation frequencies of Notch Signaling genes mapped to our data

Table S8: Mutation frequencies of Focal Adhesion genes mapped to our data

Table S9: Mutated genes of enriched pathways in all patients of ages 81 to 87

Table S9 shows the mutated genes in the four KEGG pathways found enriched in the old age group by individual patient. Genes marked in black were found in the enrichment analysis and met the 0.15 cutoff. Genes marked in red did not make the mutation frequency cutoff of 0.15 but were found mutated in single patients.

Table S10: List of genes with a significant correlation between gene mutation frequency and patient age (Spearman's rank coefficient, p-value < 0.05)
